# Finding the needle in the haystack: localization and endoscopic treatment of diverticular-associated lower GI bleeding

**DOI:** 10.1093/jcag/gwad002

**Published:** 2023-02-28

**Authors:** Fiona Milne, Robert Bechara

**Affiliations:** Division of Gastroenterology, Kingston Health Sciences Centre, Queen’s University, Kingston General Hospital, Kingston, Ontario, Canada; Division of Gastroenterology, Kingston Health Sciences Centre, Queen’s University, Kingston General Hospital, Kingston, Ontario, Canada

A 74-year-old previously healthy male presented to the Emergency Department with 12 hour history of painless hematochezia. He had positive orthostatic vitals from supine to standing. Colonoscopy was performed, which demonstrated blood clots throughout the colon without evidence of blood in the terminal ileum. Withdrawal examination was significant for sigmoid predominant diverticular disease. No other source of bleeding was identified. We irrigated the sigmoid colon with water to clear blood clots and examine the underlying diverticular disease. Careful endoscopic examination identified a diverticulum with clot within the lumen, and irrigation of the clot showed an underlying visible vessel. The vessel was clipped (Figure 1A–D).

**Figure 1.**
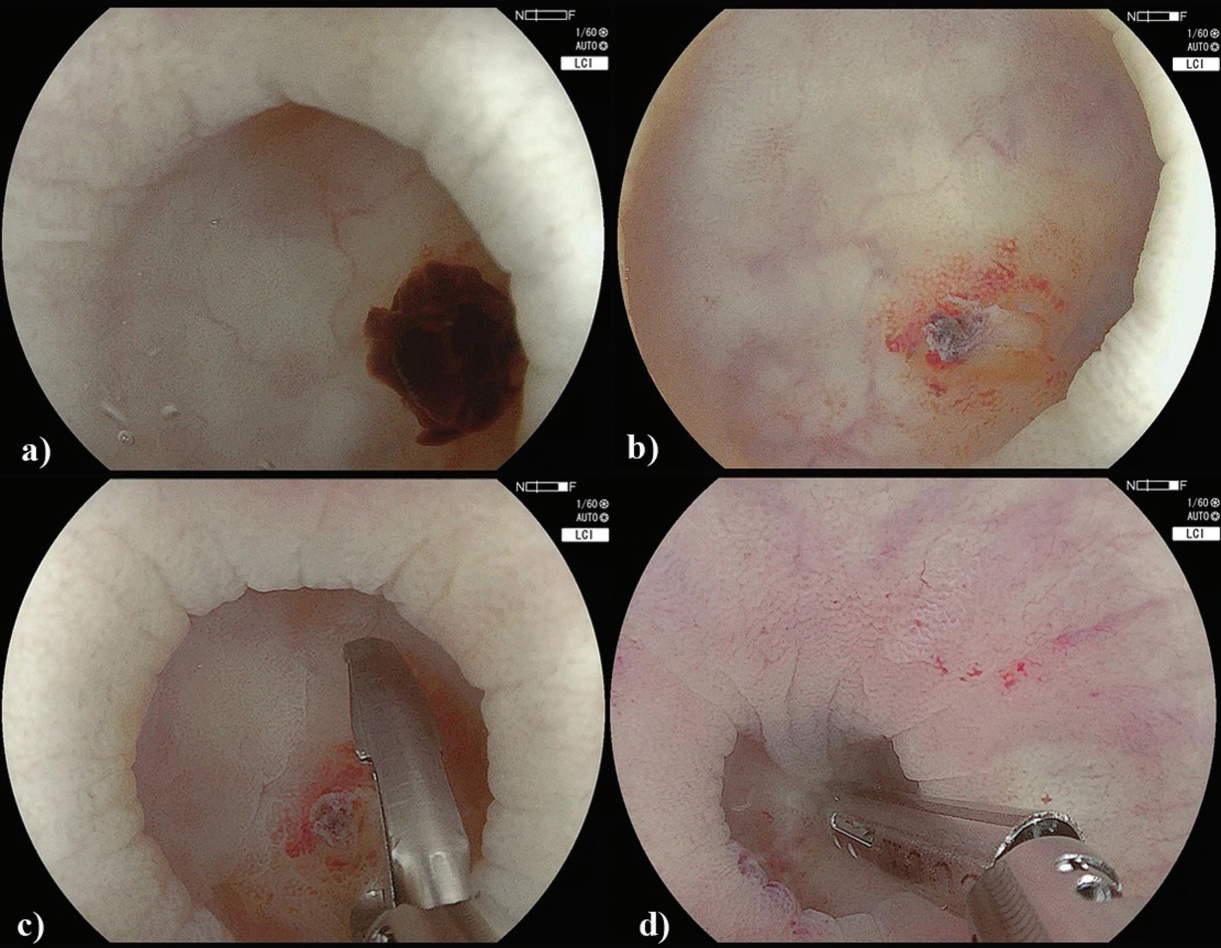
Sigmoid colon diverticulum with clot within the diverticular lumen (A), underlying visible vessel following clot clearance (B) and subsequent clipping of the visible vessel (C–D).

Diverticular bleeding accounts for approximately 20–40% of lower gastrointestinal bleeds (1). Management is generally supportive, as bleeding stops spontaneously in 70–80% of cases (2). Colonoscopy can help identify diverticula with stigmata of recent hemorrhage (SRH) such as active bleeding, visible vessels, and adherent clots, but the diagnostic yield may be as low as 20% (3). Identification of SRH can increase to 40% if colonoscopy is done early (<12 h) rather than late (48–72 h), although this does not necessarily lead to improved clinical outcomes (4). If the bleeding is localized, potential endoscopic therapies include injection, endoscopic band ligation, thermal contact, and endoscopic clipping.

## Data Availability

There are no data associated with this manuscript.

## References

[1] Hreinsson JP , Gumundsson S, Kalaitzakis E, Björnsson ES. Lower gastrointestinal bleeding: incidence, etiology, and outcomes in a population-based setting. Eur J Gastroenterol Hepatol 2013;25:37–43. doi:10.1097/MEG.0b013e32835948e3. 23013623

[2] Longstreth GF. Epidemiology and outcome of patients hospitalized with acute lower gastrointestinal hemorrhage: a population-based study. Am J Gastroenterol 1997;92:419–24. 9068461

[3] Jensen DM , Machicado GA, Jutabha R, Kovacs TO. Urgent colonoscopy for the diagnosis and treatment of severe diverticular hemorrhage. N Engl J Med 2000;342:78–82. doi:10.1056/NEJM200001133420202. 10631275

[4] Green BT , Rockey DC, Portwood G, et al. Urgent colonoscopy for evaluation and management of acute lower gastrointestinal hemorrhage: a randomized controlled trial. Am J Gastroenterol 2005;100:2395–402. doi:10.1111/j.1572-0241.2005.00306.x. 16279891

